# Impulsivity and Compulsivity After Subthalamic Deep Brain Stimulation for Parkinson’s Disease

**DOI:** 10.3389/fnbeh.2020.00047

**Published:** 2020-04-23

**Authors:** Sara Scherrer, Andrew H. Smith, Jaimie Gowatsky, Christina A. Palmese, Joohi Jimenez-Shahed, Brian H. Kopell, Helen S. Mayberg, Martijn Figee

**Affiliations:** Nash Family Center for Advanced Circuit Therapeutics, Icahn School of Medicine at Mount Sinai, New York, NY, United States

**Keywords:** Parkinson, deep brain stimulation, subthalamic nucleus, impulsivity, compulsivity

## Abstract

Impulsivity and compulsivity are prominent non-motor problems in Parkinson’s disease (PD). Despite 20 years of research, there is still an ongoing debate as to whether subthalamic deep brain stimulation (STN DBS) for PD exacerbates or improves these symptoms. Here, we review how STN DBS affects clinical symptoms and neurocognitive aspects of impulsivity and compulsivity. When comparing patients post- to pre-surgery, in the majority of studies STN DBS for PD is associated with a decrease in clinically diagnosed impulse-control disorders and disorders of compulsivity. To avoid confounds, such as post-surgical decreases in dopaminergic medication doses, comparisons can also be made between DBS “On” versus “Off” conditions. These experimentally assayed effects of STN DBS with respect to neurocognitive aspects of impulsivity and compulsivity are more mixed. STN DBS improves behavioral flexibility without impairing negative feedback learning, delay discounting, or inhibitory control, as long as stimulation is restricted to the dorsal STN. However, STN DBS may drive impulsive actions when a subject is faced with competing choices. We discuss how motivated responses may be either enhanced or impaired by STN DBS depending on engagement of dorsal or ventral STN-mediated circuits. Future studies should combine structural and functional circuit measures with behavioral testing in PD patients on and off medication and stimulation. A more sophisticated understanding of how to modulate cortico-striatal-thalamo-cortical loops will increase the likelihood that these circuit manipulation techniques can successfully be applied to a wider range of neuropsychiatric disorders.

## Introduction

Parkinson’s Disease (PD) is a neurodegenerative disorder affecting basal ganglia systems controlling motor and non-motor functions. Impulsivity and compulsivity are prevalent non-motor features of PD associated with lack of self-control. Whereas impulsivity involves diminished control over prematurely expressed actions, compulsivity refers to diminished control over repetitive, ritualistic thoughts and behaviors. In patients with PD, impulsive and compulsive symptoms can cause suffering and functional impairments, and are often unresponsive to, or even induced by, PD medications. The standard treatment for PD is dopamine replacement therapy (DRT), with either levodopa or dopamine agonists. Although these drugs are highly effective for motor symptoms such as rigidity, bradykinesia and resting tremor, they are also associated with a 2- to 3.5-fold increased odds of developing impulsive or compulsive behaviors (Weintraub et al., 2009; Santangelo et al., 2013; Kim et al., 2015).

For PD patients developing incapacitating motor fluctuations or side-effects on DRT, subthalamic deep brain stimulation (STN DBS) has proven to be an effective augmentation strategy. STN DBS, relative to DRT, may allow for less dopaminergic striatal stimulation as a result of a reduction in the total levodopa equivalent daily dose (LEDD) by an average of 73% (Lhommée et al., 2012). In theory, these medication changes may decrease the risk for impulsive and compulsive behaviors, implying that STN DBS is a beneficial option for PD patients suffering from these symptoms. However, it is possible that the stimulation is not limited to the motor subdivision of the STN, and also affects cognitive-associative and/or limbic subdivisions, which could either improve or exacerbate impulsivity and compulsivity. Indeed, the literature is conflicted regarding the effects of STN DBS on impulsivity and compulsivity in PD, with evidence for both improvement and worsening of these symptoms.

This article aims to elucidate the effects of STN DBS on impulsivity and compulsivity in PD, taking a new approach to reviewing the literature. Considering the connection of dopamine with impulsivity and compulsivity, we explore the behavioral influence of post-surgical changes in dopaminergic medication doses. In addition, we did not limit our search of the literature to inventories of clinical symptoms and diagnoses before and after surgery. Rather, we focused on changes in neurocognitive paradigms that reflect different aspects of impulsivity and compulsivity, and that can be quantified during experimental changes in stimulation delivery.

### Impulsivity

Impulse control disorders (ICDs) as classified in the Diagnostic and Statistical Manual of Mental Disorders, 5th Edition (DSM-5, American Psychiatric Association, 2013) are conditions involving problems of emotional and behavioral self-control, including intermittent explosive disorder (IED), kleptomania, pyromania, and other specified or unspecified ICDs. More commonly observed in PD are diminished control over gambling, sexual behaviors, eating, shopping, hobbyism, non-goal directed actions (punding) and medication use (Dopamine Dysregulation Syndrome, DDS) (Weintraub et al., 2009). The Questionnaire for Impulsive-Compulsive Disorders in Parkinson’s Disease Rating Scale (QUIP-RS) is the most widely used and well-validated scale for measuring likelihood and severity of ICDs in PD (Weintraub et al., 2012). As the name suggests, this scale intends to measure severity of both impulsive and compulsive disorders. Its questions therefore relate to impulsive as well as compulsive aspects of problematic behaviors, i.e., excessive urges, distressing desires, and obsessive thinking. The total score, however, is defined as severity of ICD without sub-scores for impulsive or compulsive aspects.

Complicating efforts to disambiguate impulsivity and compulsivity in commonly used scales, there has not always been consensus about the definition of impulsivity itself. Many authors agree upon a definition of impulsivity as a tendency to act prematurely with little foresight, a failure to suppress inappropriate motor, cognitive or emotional responses. This definition implies that impulsivity is a multifaceted construct requiring assessment with multiple distinct paradigms. The most widely used paradigms assess impulsivity as: (1) impaired negative feedback learning, e.g., the Iowa Gambling Task (Bechara et al., 1997) or the Probabilistic Selection Task (Frank et al., 2007); (2) a preference for small immediate reward over larger delayed reward, i.e., delayed discounting tasks (Loewenstein, 1988); (3) making responses that are premature or should be withheld, e.g., the Go/No Go Task (Ballanger et al., 2009) or Stroop test (Stroop, 1935).

### Compulsivity

The most common disorders of compulsivity are defined in the DSM-5 under Obsessive-Compulsive and Related Disorders (OCRD). These include obsessive-compulsive disorder (OCD), body dysmorphic disorder, hoarding disorder, trichotillomania (hair pulling disorder) and excoriation (skin-picking) disorder. The DSM-5 also allows for the identification of compulsions in other mental disorders, defining compulsions as repetitive behaviors or mental acts, such as hand washing, ordering, checking, praying, counting, or repeating words, which the individual feels driven to perform in response to an obsession or according to rules that must be applied rigidly. Compulsions are usually aimed at preventing or reducing anxiety, distress, or dreaded events, despite insight that the behaviors are not realistically connected to these outcomes. Severity of the prototypical disorder of compulsivity, OCD, can be measured with clinician-rated scales such the Maudsley Obsessive-Compulsive Inventory (MOCI, Hodgson and Rachman, 1977), or the Yale-Brown Obsessive-Compulsive Scale (Y-BOCS, Goodman et al., 1989). Examples of transdiagnostic compulsivity questionnaires are the DSM-5 obsessive–compulsive spectrum scale (LeBeau et al., 2013) and the Padua Inventory (Sternberger and Burns, 1990).

As a cognitive construct, compulsivity, like impulsivity, may be decomposed into various factors of a mainly cognitive, affective or motivational nature (Figee et al., 2016). First, similar to impulsivity, compulsivity implies engagement in self-defeating repetitive behaviors, which may be related to altered reward or punishment sensitivity. Second, the diminished ability to ignore or stop unwanted ideas or actions suggests the presence of cognitive or behavioral inflexibility, as measured with reversal-learning tasks (i.e., Wisconsin Card Sorting Task, Grant and Berg, 1948) or attentional set-shifting tasks (i.e., Trail Making Task, Ardouin et al., 1999). Third, habitual responding and diminished goal-directed activity suggests excessive habit-learning, as measured with instrumental or model-based learning paradigms (Daw et al., 2005). However, firm consensus about the definition of compulsivity as a cognitive construct remains to be established. Further, paradigms which explicitly test compulsivity are difficult to develop because compulsive behaviors are often triggered by person-specific circumstances that are challenging to recreate in generalizable tasks. In addition, there may be overlap between impulsivity and compulsivity particularly in reinforcement learning paradigms. Nevertheless, paradigms testing cognitive and behavioral flexibility and habit-learning tap into important aspects of compulsivity which we use in the present review.

## Methods

### Search Strategy

We conducted a literature search using the Pubmed database for articles published between January 1st 2002 and June 27th 2019. Keywords for impulsivity associated with subthalamic DBS in PD were based on ICDs, associated scales, and neurocognitive paradigms:

Subthalamic DBS, Parkinson, Impulsivity, Impulse control disorder, Intermittent Explosive Disorder, Kleptomania, pyromania, gambling, sexual behaviors, eating, shopping, hobbyism, punding, Dopamine Dysregulation syndrome, QUIP-RS, extinction learning, reward choice, response inhibition, Barratt Impulsiveness Scale, The Iowa Gambling Task, Instrumental Learning Task, The Game of Dice Task, The Temporal Discounting Task, Deal or No Deal, Cambridge Gambling Task, The Probabilistic selection task, *Status Quo* Task, Auditory two-alternative forced choice task, The Go/No-Go task, The Stroop Test.

Similarly, for compulsivity we used the following keywords:

Subthalamic DBS, Parkinson, compulsivity, Obsessive-Compulsive Disorder (OCD), OCRD, body dysmorphic disorder, hoarding, trichotillomania, excoriation, hand washing, ordering, checking, praying, counting or repeating words, Maudsley Obsessive-Compulsive Inventory, Yale-Brown Obsessive-Compulsive Scale, Obsessive–Compulsive Spectrum Scale, Padua Inventory, reward punishment sensitivity, cognitive inflexibility, habit learning, probabilistic reversal-learning, attentional set-shifting tasks, habit-learning, devaluation, Trail Making Task, Wisconsin Card Sorting Task, Probabilistic classification task.

After the initial search, a reference analysis was conducted to find additional reports. We excluded studies published before 1999, reviews, animal studies, and computational modeling studies. Only studies investigating the effects of bilateral STN DBS in PD patients were included. Clearly defined assessment tools and dopaminergic medication status at time of testing had to be described. Reports in languages other than English, or that did not include statistical tests, were excluded.

### Impulsivity Measures

We searched for studies reporting ICDs related to STN-DBS in PD, and for studies measuring DBS-related impulsivity with the following scales or paradigms:

Barratt Impulsiveness Scale (BIS-11, Patton and Stanford, 1995): a 30-item questionnaire designed to assess impulsive personality traits, with sub-scores for attentional impulsivity, motor impulsivity and non-planning impulsivity.

Negative Feedback Learning (Table 1): Iowa Gambling Task (IGT, Bechara et al., 1997), Game of Dice Task (GDT, Brand et al., 2005), Instrumental Learning Task (ILT, Seymour et al., 2016), and Probabilistic Selection Task (Frank et al., 2004, 2007). These paradigms may be used for measuring impulsivity defined as impaired learning from negative feedback. In the IGT and GDT, participants have to balance their choices between safe and advantageous options (card decks or dice rolls with low reward and small losses) and risky and disadvantageous options (high rewards and high losses). In the ILT, participants choose from four abstract pictures based on feedback from previous trials where their choice was either rewarded (receiving tokens) or punished (losing tokens). In all of these tasks, impulsive participants may insufficiently learn from previous loss-trials or series. A probabilistic selection task may also be used to measure impulsivity defined as impaired learning from negative feedback, or (see below) as premature responding under high-conflict conditions (when presented with conflicting positive reward probabilities). Impulsive participants may insufficiently learn from their previous losses and/or respond faster in high-conflict trials.

**TABLE 1:** Negative feedback learning.

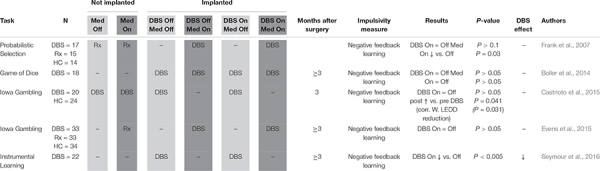

*Impulsivity paradigm employed (Task) shown for each study. Number of subjects is given for each group: Parkinson’s Disease (PD) patients who underwent DBS, PD patients treated only with medications (Rx), and healthy controls (HC). Distribution of PD cases across different conditions are given. Also provided is number of months between surgery and evaluation, impulsivity construct utilized, and experimental results. ↑ denotes improvement in performance and ↓ denotes impairment, and – denotes no change. For ease of interpretation, effects of STN DBS activation in experimental sessions are highlighted in a separate column.*

Delay Discounting (Table 2): Delay discounting tasks may be used to measure impulsivity defined as discounting of larger delayed reward over smaller immediate reward. Examples include the Kirby Delay-Discounting Task (Kirby and MarakoviĆ, 1996). Impulsive participants may impulsively discount future reward, i.e., show increased delay discounting. The Cambridge Gambling Task (Rogers et al., 1999), used by Torta et al. (2012), measures delay aversion, identifying subjects who repeatedly pick the initial bet offered, and results are here presented alongside studies of delay discounting, the measure of impulsive tendencies it most closely resembled.

**TABLE 2:** Delay discounting.

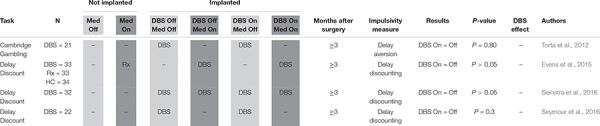

*Impulsivity paradigm employed (Task) is shown for each study. Number of subjects is given for each group: Parkinson’s Disease (PD) patients who underwent DBS, PD patients treated only with medications (Rx), and healthy controls (HC). Distribution of PD cases across different conditions are given. Also provided is number of months between surgery and evaluation, impulsivity construct utilized, and experimental results. ↑ denotes improvement in performance and ↓ denotes impairment, and – denotes no change. For ease of interpretation, effects of STN DBS activation in experimental sessions are highlighted in a separate column.*

Inhibitory control (Table 3): Go/No-Go task (Donders, 1969), Stop Signal Task [SST, (Logan and Cowan, 1984)], *Status Quo* Task (SQT, Fleming et al., 2010) and Stroop test (Stroop, 1935), Simon (Simon and Rudell, 1967). These tasks may be used to measure impulsivity defined as impaired inhibitory control over prepotent motor or cognitive responses. In Go/No-Go, SST and SQT, participants are presented with a stimulus that requires them either to respond (Go) or withhold a response (No-Go/Stop). Impulsive participants may make more commission errors (Go responses on No-go or Stop trials) and/or anticipation errors (responding too fast). SQT is a modified Go/No-Go measuring response inhibition under high conflict. A ball appears either between two lines (Go) or outside the lines (No-Go). High conflict occurs when the ball is almost touching the line. In the Stroop test, words representing colors are written in colors incongruent to the written word, and participants are required to state aloud the ink color as opposed to reading the written word. Impulsive participants may make more commission errors (reporting the word instead of the color). In the Simon task participants respond to visual cues prompting either contra- or ipsilateral movements.

**TABLE 3:** Inhibitory control.

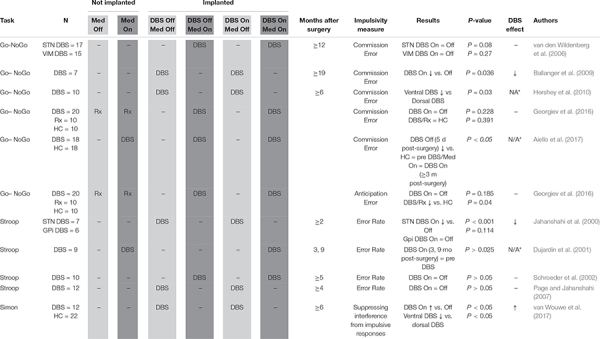

Impulsivity paradigm employed (Task) is shown for each study. Number of subjects is given for each group: Parkinson’s Disease (PD) patients who underwent DBS, PD patients treated only with medications (Rx), and healthy controls (HC). Distribution of PD cases across different conditions are given. Also provided is number of months between surgery and evaluation, impulsivity construct utilized, and experimental results. ↑ denotes improvement in performance and ↓ denotes impairment, and – denotes no change. For ease of interpretation, effects of SNT DBS activation in experimental sessions are highlighted in a separate column. Sub-thalamic nucleus (STN), ventral intermediate nucleus of the thalamus (VIM), globus pallidus interna. (Gpi), not applicable (N/A). All DBS experiments involved STN DBS unless specified otherwise. *No *P-*value from an experimental session comparing Stimulation On vs. Stimulation Off.

Responding Under High Conflict (Table 4): ILT, Probabilistic selection tasks, delay discounting, and SQT, discussed above, can all be utilized to gauge impulsive behavior under so called “high conflict” scenarios. These are situation where the range of options available for selection are less readily distinguished from one another. Other paradigms have also been used for this purpose, such as an auditory forced choice task (London et al., 2019).

**TABLE 4:** Responding under high conflict.

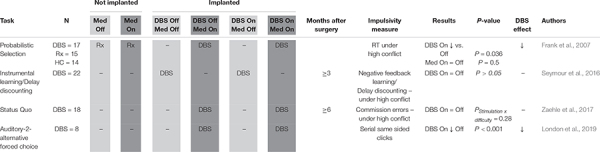

*Impulsivity paradigm employed (Task) is shown for each study. Number of subjects is given for each group: Parkinson’s Disease (PD) patients who underwent DBS, PD patients treated only with medications (Rx), and healthy controls (HC). Distribution of PD cases across different conditions are given. Also provided is number of months between surgery and evaluation, impulsivity construct utilized, and experimental results. ↑ denotes improvement in performance and ↓ denotes impairment, and – denotes no change. For ease of interpretation, effects of STN DBS activation in experimental sessions are highlighted in a separate column. Reaction time (RT).*

### Compulsivity Measures

In addition to studies reporting the prevalence of OCD and OCRD before and after STN DBS in PD, we searched for studies measuring OCD-severity using the Maudsley Obsessive-Compulsive Inventory (MOCI, Hodgson and Rachman, 1977) or the Yale-Brown Obsessive- Compulsive Scale (Y-BOCS, Goodman et al., 1989). The MOCI is a self-rated 30-item scale with sub-scores for checking, cleaning, slowness, and doubting. The Y-BOCS is a clinician-rated 10-item scale with sub-scores for obsessions and compulsions.

In addition, we searched for studies examining aspects of compulsivity in PD related STN DBS utilizing one or more of the following paradigms:

Habit learning tasks (Daw et al., 2005; Gillan et al., 2011) measure compulsivity defined as the dominance of habits over goal-directed learning. Participants learn to respond to stimuli with rewarding or negative reinforcing outcomes which are eventually devalued. Compulsive participants keep habitually responding to devalued stimuli, or show more model-free learning (predicted by reinforcement history) than model-based learning (adapted to devaluation).

Perseveration (Table 5): Wisconsin Card Sorting Task (WCST, Grant and Berg, 1948) is a reversal learning task which may be used to measure compulsivity defined as cognitive inflexibility. Cards must be sorted on the basis of either number, form or color with the sorting rule alternating after 10 correct responses, a fact learned by the participant through trial and error. Compulsive participants make more perseverative errors, indicated by two successive sorts on an incorrect dimension. The original Wisconsin Card Sorting Task (WCST) uses 128 response cards, and a Modified Wisconsin Card Sorting Task (MWCST) simplifies the task, using only 48 response cards (Nelson, 1976).

**TABLE 5:** Perseveration.

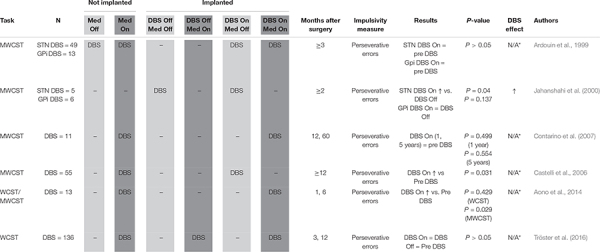

*Compulsivity paradigm employed (Task) is shown for each study. Number of subjects is given for each group: Parkinson’s Disease (PD) patients who underwent DBS. Distribution of PD cases across different conditions are given. Also provided is number of months between surgery and evaluation, impulsivity construct utilized, and experimental results. ↑ denotes improvement in performance and ↓ denotes impairment, and – denotes no change. For ease of interpretation, effects of STN DBS activation in experimental sessions are highlighted in a separate column. Wisconsin Card Sorting Task (WCST), Modified Wisconsin Card Sorting Task (MWCST), sub-thalamic nucleus (STN), globus pallidus interna (GPi). ^∗^No *P-*value from an experimental session comparing Stimulation On vs. Stimulation Off.*

Attentional set shift (Table 6): Trail Making Task (TMT) Part B (Corrigan and Hinkeldey, 1987) may be used to measure compulsivity defined as impaired attentional set-shifting. Participants must draw a line alternating between letters and numbers (A-1-B-2-C-3, etc.). Compulsive individuals tend to have longer completion times due to less attentional flexibility.

**TABLE 6:** Attentional set-shifting.

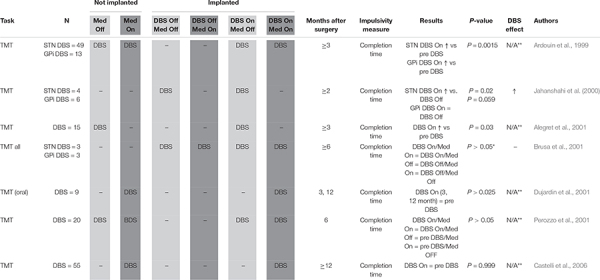

Compulsivity paradigm employed (Task) is shown for each study. Number of subjects is given for each group: Parkinson’s Disease (PD) patients who underwent DBS. Distribution of PD cases across different conditions are given. Also provided is number of months between surgery and evaluation, impulsivity construct utilized, and experimental results. ↑ denotes improvement in performance and ↓ denotes impairment, and – denotes no change. For ease of interpretation, effects of SNT DBS activation in experimental sessions are highlighted in a separate column. Trail making task (TMT), sub-thalamic nucleus (STN), globus pallidus interna (GPi). ^∗^STN and GPi patients were jointly analyzed. **No *P-*value from an experimental session comparing Stimulation On vs. Stimulation Off.

## Results

### Impulse Control Disorders

We found 12 studies investigating the prevalence of ICDs (gambling, compulsive shopping, binge eating, hypersexuality, punding, DDS) in a total of 582 PD patients pre- and (6–12 months) post STN DBS (Ardouin et al., 1999; Lim et al., 2009; Lhommée et al., 2012; Shotbolt et al., 2012; Eusebio et al., 2013; Kim et al., 2013; Amami et al., 2015; Castrioto et al., 2015; Gee et al., 2015; Merola et al., 2017). The combined prevalence of ICDs in these patients was 28% (*N* = 162) before STN DBS and 6% (*N* = 32) after STN DBS (with variable post-surgical LEDD decreases). In the 162 patients that already showed ICDs before DBS, post-surgical improvement of ICDs was observed in 86% (139 patients), including full remission in 68%. Worsening of ICDs after STN-DBS was found in only 3% of all patients (Pallanti et al., 2010; Lhommée et al., 2012). Although new onset of ICDs after STN-DBS was reported in 38 patients, most of these cases were transient (Shotbolt et al., 2012; Kim et al., 2013; Amami et al., 2015). Factors that were associated with ICDs after DBS were personality disorders, dyskinesias and higher post-surgical LEDD.

### Impulsivity

#### Clinical Scales

Impulsivity scores on the Barratt Impulsiveness Scale (BIS-11, Patton and Stanford, 1995) were measured in six studies (total of 165 patients) comparing scores either between DBS On and Off conditions (Torta et al., 2012; Seinstra et al., 2016), or between DBS On and PD-patients On medication or healthy controls (Hälbig et al., 2009; Evens et al., 2015; Hagelweide et al., 2018; Irmen et al., 2019). One study reported significantly worse impulsivity scores in a DBS-group compared to healthy controls (Hälbig et al., 2009). For all other studies, no differences in BIS-11 impulsivity scores were found between DBS On and control groups.

#### Negative Feedback Learning

Impulsivity defined as impaired negative feedback learning was measured in five studies in a total of 95 PD STN DBS patients (Table 1; Frank et al., 2007; Boller et al., 2014; Castrioto et al., 2015; Evens et al., 2015; Seymour et al., 2016). An early study using a probabilistic selection task reported that negative feedback learning was impaired by dopaminergic medication in PD patients without DBS, but not by DBS activation in PD patients who had been implanted (Frank et al., 2007). While a subsequent study using the GDT to evaluate negative feedback learning in implanted patients did not replicate the deleterious effect of dopaminergic medication (Boller et al., 2014), a different study called attention to the much lower doses of dopaminergic medication used post-implantation, and showed a relationship between post-implantation medication dose decreases and recovery of negative feedback learning as measured by the IGT (Castrioto et al., 2015). Both of these two studies (Boller et al., 2014; Castrioto et al., 2015), as well as a separate study using the IGT (Evens et al., 2015), replicated that initial finding (Frank et al., 2007) that DBS does not impair negative feedback learning. Only one study (Seymour et al., 2016), employing an ILT, suggested decreased negative feedback learning secondary to DBS activation. In conclusion, the reviewed data suggest that dopaminergic medication increases impulsivity defined as impaired negative feedback learning, but STN DBS does not. Although negative feedback learning may even improve after STN DBS, this appears to be primarily related to the post-surgical decrease of dopaminergic medication.

#### Delay Discounting

Four studies measured DBS-related delay discounting in a total of 108 PD patients (Table 2; Torta et al., 2012; Evens et al., 2015; Seinstra et al., 2016; Seymour et al., 2016). None of these studies found evidence that DBS activation interferes with subjects’ ability to delay claiming a reward in order to maximize total reward, suggesting that DBS does not affect this aspect of impulsivity.

#### Inhibitory Control

Impulsivity defined as premature responding to stimuli was measured in a total of 130 STN-DBS PD patients (Table 3; Jahanshahi et al., 2000; Dujardin et al., 2001; Schroeder et al., 2002; van den Wildenberg et al., 2006; Page and Jahanshahi, 2007; Ballanger et al., 2009; Hershey et al., 2010; Georgiev et al., 2016; Aiello et al., 2017). An early study (van den Wildenberg et al., 2006) was not able to show a significant difference (*P* = 0.08) between DBS On and Off conditions when examining rate of NoGo commission errors. However, two subsequent studies (Ballanger et al., 2009; Hershey et al., 2010) did show that stimulation impaired inhibitory control, making subjects respond to NoGo signals more frequently. Notably, Hershey et al. (2010) used an innovative strategy of recruiting only subjects who had STN electrodes placed at different depths on contralateral sides, enabling them to fine map the anatomy most relevant to impulse control. Specifically, they identified the ventral STN as most relevant to impaired inhibition, consistent with the ventral STN’s closer connection to association cortex compared to the dorsal STN, which is thought to be more closely connected to sensorimotor cortex.

Authors employing the Simon task also found evidence for the functional relevance of a ventral-dorsal STN axis, namely that dorsal stimulation leads to more inhibitory capacity compared to ventral territory stimulation (van Wouwe et al., 2017). Georgiev et al. (2016) were unable to confirm higher rates of NoGo commission errors during STN stimulation, and also did not observe increases in premature responding (anticipation errors). One study showed significant impairment in NoGo paradigm response inhibition prior to DBS being switched on (Aiello et al., 2017), but these subjects were evaluated only 5 days post-surgery, making it unclear whether acute post-operative factors contributed to cognitive deficits. Jahanshahi et al. (2000) showed a heightened error rate on the Stroop test, which was specific to STN stimulation, being unaffected by globus pallidus interna (GPi) stimulation. Two subsequent studies (Schroeder et al., 2002; Page and Jahanshahi, 2007) were unable to replicate this detrimental impact on Stroop error rate. Dujardin et al. (2001) looked at Stroop performance pre- and post-surgery, but did not experimentally compare DBS On and Off conditions. In conclusion, although initial small studies report stimulation-related impaired inhibitory control, subsequent studies in larger samples showed no deleterious effect of stimulation, or even improved inhibitory control, especially with stimulation of the dorsal STN.

#### Responding Under High Conflict

Impulsive responding under high conflict scenarios was measured in four studies in a total of 65 STN DBS patients (Table 4; Frank et al., 2007; Zaehle et al., 2017; London et al., 2019). Frank et al. (2007) and Seymour et al. (2016), already discussed above, were able to utilize their respective paradigms to assess whether exacerbation of impulsivity by STN DBS could be more easily appreciated under conditions of high conflict. Only Frank et al. (2007) observed a significant effect, with the DBS On condition characterized by an impairment in the ability of subjects to slow down during situations requiring more careful evaluation. Along similar lines, Zaehle et al. (2017) looked for a stimulation-difficulty interaction on “false alarm” rates during the SQT, but reported a non-significant interaction term. In contrast, London et al. (2019) who assigned each subject “impulsivity indexes” based on sequential same-sided clicks in an auditory two-alternative forced choice task, did report a positive finding. Specifically, they showed that stimulation increased the impulsivity index only during trials with higher levels of conflict. Together, these studies suggest that STN DBS negatively affects impulse control under high conflict conditions.

### Disorders of Compulsivity

Subthalamic deep brain stimulation for OCD gained attention after two patients were treated with STN DBS for PD with comorbid OCD (Mallet et al., 2002). Both cases experienced improvement in motor as well as obsessive-compulsive symptoms. Two weeks after the procedure, the patients were free from compulsions, and obsessions improved by 58% in patient 1 and by 64% in patient 2. Since then, the literature on co-morbid OCD and OCDR in patients with PD undergoing STN DBS is surprisingly sparse.

### Compulsivity

#### Clinical Scales

Only two studies (Alegret et al., 2001; Hälbig et al., 2009) used obsessive-compulsive symptom scales during STN DBS for PD, both using the MOCI. One of these studies administered the MOCI pre and post STN DBS (*n* = 15), finding that STN DBS improved obsessive-compulsive traits. The pre-surgical score average of 8.40 decreased significantly at the 3 months follow-up, down to 5.47 (Alegret et al., 2001). A second study reported no difference in MOCI scores when making an intersubject comparison between 16 STN PD patients (*M* = 6.8, *SD* = 2.59) and 37 PD patients treated only with medication (*M* = 6.79, *SD* = 3.85) (Hälbig et al., 2009). No studies were found that administered the Y-BOCS in Parkinson’s patients treated with STN DBS.

#### Habit Formation

We were unable to find studies that applied habit-learning paradigms in STN DBS PD patients. However, we did find studies measuring compulsivity defined as impaired perseveration or attentional set-shifting (see below).

#### Perseveration

A total of six publications were reviewed which evaluated the effects of STN DBS on compulsivity in a total of 271 subjects using a perseveration paradigm (Table 5), which aims to measure persistence of unrewarded responses and inability to update decision rules. All protocols utilized the WCST or the MWCST. Rates of perseverative errors, reflecting inappropriate continued employment of ineffective selection strategies, are considered to reflect cognitive inflexibility. Isolating the behavioral rigidity common in individuals with compulsive behaviors and disorders, this metric has been widely employed to evaluate one facet of compulsivity in the STN DBS population.

Upon review of perseverative error rates reported in these studies, it appears that, overall, STN DBS has no deleterious effect on, if not improves, this index of compulsivity. Three studies (Ardouin et al., 1999; Contarino et al., 2007; Tröster et al., 2016) found no significant effect of STN DBS on perseverative errors. All three of these studies used a within-subjects design, comparing patients before and after device implantation. The study by Tröster et al. (2016) differed in that a subset of patients were randomized to delayed device activation, and the authors used the original WCST instead of the less ambiguous MWCST (Nelson, 1976). Another study comparing pre- and post-operative subjects (Aono et al., 2014) also found no difference when using the WCST, but did find significant performance improvement on the MWCST. Castelli et al. (2006) also reported significant improvement on the MWCST post-surgery. Only one study (Jahanshahi et al., 2000) experimentally assessed the effects of stimulation by conducting testing both with the stimulator on and with the stimulator switched off. These authors found that STN stimulation, but not GPi stimulation, decreased the number of MWCST perseverative errors.

#### Attentional Set-Shifting

Another means of quantifying inflexibility is via measuring ability to shift attention. Seven studies with a total of 155 STN DBS patient (Ardouin et al., 1999; Jahanshahi et al., 2000; Alegret et al., 2001; Brusa et al., 2001; Dujardin et al., 2001; Perozzo et al., 2001; Castelli et al., 2006) deployed the TMT, measuring time to completion (Table 6). Shorter time to completion was taken to reflect less rigid and more flexible cognition. Two early studies (*N* = 49 and 15, respectively) compared STN DBS patient pre- and post-implantation and found improvement, with patients able to complete the task more quickly once DBS had begun (Ardouin et al., 1999; Alegret et al., 2001). Two other early studies with a smaller combined sample size (*N* = 9 and 20, respectively) using a similar pre-/post-implantation design did not find a significant effect (Dujardin et al., 2001; Perozzo et al., 2001), nor did a later study assessing 55 patient pre- and post-implantation (*N* = 55). Two studies used controlled experimental designs, but had smaller samples sizes, reflecting the greater difficulty of conducting such studies. The first (Jahanshahi et al., 2000), found that stimulation significantly shortened TMT completion time in four STN DBS patients, while the second (Brusa et al., 2001) detected only a non-significant decrease in mean completion time when stimulation was switched on in a mixed sample of STN and GPi DBS patients off medication (Brusa et al., 2001).

## Discussion

To date, studies in DBS PD patients have employed a wide variety of strategies to dissect out the specific behavioral relevance of the STN, a key relay station in cortico-stiatal-thalamo-cortico (CSTC) loops. The sheer diversity of approaches, both conceptually and experimentally, is evident from the above review, and the range of paradigms can make it difficult to discern larger patterns in the literature. Twenty years of work on DBS in PD has, though, produced a number of findings consistent with a facilitation of cognitive-behavioral flexibility alongside restoration of purposeful movements, although possibly at the cost of some diminished capacity for behavioral inhibition especially under high-conflict conditions.

Subthalamic deep brain stimulation for PD is thought to exert its primary effect by restoring the balance of activity between direct and indirect pathways (Jakobs et al., 2019). Early studies reinforced concerns that damage to the integrity of these pathways in PD could lead to an unwanted increase in impulsive and compulsive behaviors when DBS was applied. However, when evaluating the extant literature on this topic, we found that the majority of studies identified a decrease in ICDs after STN DBS. Nevertheless, there are important caveats. ICDs are binary clinical diagnoses based on the detrimental impact of behavioral patterns on social functioning over time, which means they are not experimentally tractable constructs. Evidence about ICDs is therefore limited to pre- and post-surgery comparisons, as opposed to laboratory manipulations. However, pre- and post-comparisons are subject to confounds, such as post-operative LEDD decreases (Castrioto et al., 2015). Dopaminergic medications are associated with ICDs and DDS (Castrioto et al., 2015), and dose reductions (a major goal of DBS surgery) may be the predominant factor influencing changes in ICD prevalence, leading to a dramatic decrease post-surgery (5%) compared to pre-surgery (28%). Avoiding such confounds was an important motivation behind our prioritizing identification of non-binary measures such as scales (e.g., the BIS) and cognitive-behavioral constructs (e.g., delay discounting) more amenable to controlled evaluation via electrode current manipulation. This approach revealed the value of targeting different sub-domains of impulsivity, with different tasks yielding different findings: (1) The evidence suggests that negative feedback learning is impaired by dopaminergic medication but not by STN DBS. (2) We found no evidence that delay discounting tendencies are exacerbated by STN DBS. (3) With response inhibition, the picture is more mixed. Earlier studies suggest that STN DBS may impair inhibitory control. However, later studies suggest that the effects on inhibitory control may be dependent on the location of stimulation, with impairments after ventral stimulation but improvements after dorsal stimulation. (4) Finally, in so called “high conflict” scenarios, where a greater degree of ambiguity is built into the patient’s task using more involved study designs, we identified evidence of heightened impulsivity after STN DBS.

**FIGURE 1:**
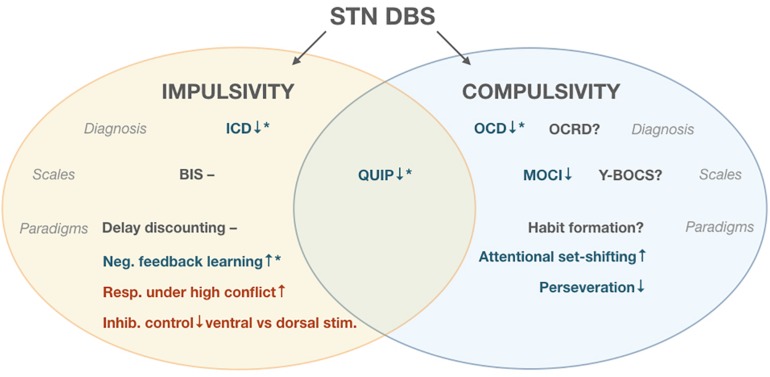
Effects of Subthalamic Deep Brain Stimulation (STN DBS). **↑**denotes increase in measured parameter, ↓denotes decrease, and – denotes no change. 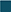 indicates improvement, 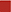 indicates impairment * post-surgical decrease in levodopa equivalent daily dose (LEDD), ? indicates measure unreported in literature. Impulse Control Disorders (ICD), Obsessive Compulsive Disorder (OCD), Obsessive Compulsvie Related Disorders (OCRD), Barrat Impulsiveness Scale (BIS), Questionnaire for Impulsive-Compulsive Disorders (QUIP), Maudsley Obsessional-compulsive inventory (MOCI), Yale-Brown Obsessive Compulsive Scale (Y-BOCS).

Studies of compulsivity-related diagnoses and measures during STN DBS for PD were much sparser than probes of impulsivity. For unclear reasons, studies of compulsivity were also less likely to report experimental current manipulations than pre-/post-surgery comparisons, which as discussed above are subject to confounds. One study (Alegret et al., 2001) found a decrease in OCD severity (MOCI) scores post-STN electrode activation. In terms of specific facets of compulsivity, only one study experimentally quantified perseverative error rates in DBS-On vs. –Off conditions (Jahanshahi et al., 2000), finding that stimulation decreased perseverative errors. Similarly, only two studies compared set shifting under On vs. Off conditions, with one finding a significant increase in flexibility (Jahanshahi et al., 2000), and one finding a non-significant improvement (Brusa et al., 2001). Thus, although all three of the identified experimental studies were directionally consistent with diminished compulsivity during DBS, the extremely limited total sample size warrants substantial caution in drawing generalized conclusions. Further support for the relevance of the STN to compulsivity comes from the literature on STN DBS for treatment of OCD in non-PD patients. Although this body of evidence is beyond the scope of the current review, in line with our summary of ventral territory-specific stimulation effects on impulsivity, amelioration of OCD symptoms has generally been attempted via use of more ventral electrode contacts within the STN (Mallet et al., 2008) (Figure 1).

The results discussed in this review raise the possibility that there may be benefits as well as trade-offs inherent in modulating the balance between the action suppressing and promoting functions of CSTC loops. Dampening of suppressive STN (indirect) pathways may enhance behavioral flexibility, but at the cost of impaired self-control when faced with competing choices. STN DBS is able to normalize motor control by reducing excessive beta band activity in STN and motor cortex (Whitmer et al., 2012). However, appropriate motor responses when faced with competing choices may also require coherence of theta and delta bands between the STN and medial prefrontal cortex, to facilitate the appropriate delay of motivated responses (Zavala et al., 2014). The reviewed data suggest that STN DBS may facilitate these effects in a circuit-specific manner, with dorsal stimulation being associated with less impulsivity relative to ventral stimulation. The dorsal STN shows connectivity with (pre-) motor and prefrontal motor control areas, whereas the more ventral STN is linked to limbic circuits regulating motivational control. This suggests that for impulsive PD patients, stimulation should probably be restricted to dorsal STN-pathways. However, symptoms suggestive of motivational deficits such as apathy or depression, were recently found to improve with stimulation of ventral limbic pathways (Petry-Schmelzer et al., 2019). Optimizing non-motor outcomes during STN DBS may therefore depend on reversing patient-specific imbalances in distinct motor and motivational circuits.

Efforts to learn about impulsivity and compulsivity via STN DBS in PD patients rely on convenience samples, and while they present unique opportunities to causally test hypotheses in humans, there are important limitations that must be borne in mind. First, it is not possible to test specificity of regional effects, since it would be ethically problematic to place electrodes into brain regions without a reasonable amount of prior evidence to suggest clinical benefit. This limitation is not present in the animal literature, where circuit manipulations may be performed in line with principles of experimental design. Indeed, early rodent studies of experimental STN lesions demonstrated an increase in premature responding during the 5-choice serial reaction time test, although there was also evidence of increased perseveration (Baunez et al., 2011). More recent work in rodents examining repeated sessions of STN high frequency stimulation has also shown premature responding during measurement of impulse control (Aleksandrova et al., 2013). Experiments performed in model systems have also pointed to mechanistic hypotheses about DBS beyond straightforward inactivation of STN neurons, such as possible facilitation of GABAergic STN efferents (Windels et al., 2000). Second, all implanted subjects were diagnosed with PD, and have CSTC loops that have already been forced to adapt to the characteristic neuropathological changes that underlie PD. In contrast to non-invasive neuromodulation technologies, it is not possible to enroll healthy controls. Third, we did not review the effects of DBS on other domains that are relevant for disorders of impulsivity and compulsivity, such as reward sensitivity, or risk tolerance. Fourth, in an effort to characterize particular aspects of impulsivity and compulsivity, a long list of investigator-specific tasks has been developed and refined. These task differences limit the applicability of wide-spread meta-analysis statistical techniques that rely on carefully harmonized phenotypes, and illustrate the value of ongoing NIMH initiatives to promote common data elements (Barch et al., 2016). The ideal set-up for disentangling the causal effects of disease, dopaminergic medication, surgery, and stimulation would be to prospectively evaluate pre-surgical impulsivity and compulsivity on and off medication in a large cohort of patients using a common set of measures, followed by post-surgical evaluations on and off medication and DBS. However, no study to date has applied this design. In addition, further investigation is needed into how deliberate positioning of electrode contacts at different points along the STN axes may differentially affect impulsivity and compulsivity (Mallet et al., 2007).

## Conclusion

In conclusion, despite small samples sizes, logistical challenges, and methodological heterogeneity, the evidence reviewed here tentatively suggests that dampening of suppressive STN-mediated pathways in PD decreases the risk of ICDs and compulsivity-related diagnoses (via dopaminergic medication dose decreases) and enhances cognitive-behavioral flexibility, but at the cost of impaired self-control when faced with competing choices. We need a much fuller understanding of structural and functional circuits encompassing the STN, and of how DBS and dopaminergic medication can optimally interact with these circuits to alleviate motor and non-motor symptoms in PD. This needed advance could be achieved by combining electroencephalography and tractography with behavioral testing on and off medication and stimulation in prospective PD DBS cohorts. Progress on this front is a prerequisite for application of human circuit manipulation technologies to a wider range of debilitating neuropsychiatric disorders involving disordered behavior.

## Author Contributions

SS, AS, and MF contributed to the conception and design of the review and wrote the first draft of the manuscript. SS, JG, and MF performed the search and reviewed all articles. All authors contributed to manuscript revision, and read and approved the submitted version.

## Conflict of Interest

JJ-S has received consulting fees from Boston Scientific, Abbott and Medtronic, and research support from Abbott and Medtronic. BK has received consulting fees from Abbott, Medtronic and Blackrock Therapeutics. HM has received consulting and intellectual property licensing fees from Abbott Labs Neuromodulation (previously St. Jude Medical Inc.). The remaining authors declare that the research was conducted in the absence of any commercial or financial relationships that could be construed as a potential conflict of interest.
